# Increased placental expression and maternal serum levels of apoptosis-inducing *TRAIL* in recurrent miscarriage

**DOI:** 10.1016/j.placenta.2012.11.032

**Published:** 2013-02

**Authors:** K. Rull, K. Tomberg, S. Kõks, J. Männik, M. Möls, M. Sirotkina, S. Värv, M. Laan

**Affiliations:** a Human Molecular Genetics Group, Institute of Molecular and Cell Biology, University of Tartu, Riia St. 23, Tartu 51010, Estonia; b Department of Obstetrics and Gynecology, University of Tartu, Tartu 51010, Estonia; c Department of Physiology, University of Tartu, Tartu 50411, Estonia; d Institute of Mathematical Statistics, University of Tartu, Tartu 50409, Estonia; e Department of Pathology, Tartu University Hospital, Tartu 50411, Estonia; f Department of Cell Biology, Institute of Molecular and Cell Biology, University of Tartu, Tartu 51010, Estonia

**Keywords:** Recurrent miscarriage, Whole-genome placental gene expression, *TRAIL*, *S100A8*, Maternal serum biomarker for early pregnancy failure

## Abstract

**Introduction:**

Recurrent miscarriage (RM; ≥3 consecutive pregnancy losses) occurs in 1–3% of fertile couples. No biomarkers with high predictive value of threatening miscarriage have been identified. We aimed to profile whole-genome differential gene expression in RM placental tissue, and to determine the protein levels of identified loci in maternal sera in early pregnancy.

**Methods:**

GeneChips (Affymetrix^®^) were used for discovery and Taqman RT-qPCR assays for replication of mRNA expression in placentas from RM cases (*n* = 13) compared to uncomplicated pregnancies matched for gestational age (*n* = 23). Concentrations of soluble *TRAIL* (sTRAIL) and calprotectin in maternal serum in normal first trimester (*n* = 35) and failed pregnancies (early miscarriage, *n* = 18, late miscarriage, *n* = 4; tubal pregnancy, *n* = 11) were determined using ELISA.

**Results:**

In RM placentas 30 differentially expressed (with nominal *P*-value < 0.05) transcripts were identified. Significantly increased placental mRNA expression of TNF-related apoptosis-inducing ligand (*TRAIL*; *P* = 1.4 × 10^−3^; fold-change 1.68) and *S100A8* (*P* = 7.9 × 10^−4^; fold-change 2.56) encoding for inflammatory marker calprotectin (S100A8/A9) was confirmed by RT-qPCR. When compared to normal first trimester pregnancy (sTRAIL 16.1 ± 1.6 pg/ml), significantly higher maternal serum concentration of sTRAIL was detected at the RM event (33.6 ± 4.3 pg/ml, *P* = 0.00027), and in pregnant women, who developed an unpredicted miscarriage 2–50 days after prospective serum sampling (28.5 ± 4.4 pg/ml, *P* = 0.039). Women with tubal pregnancy also exhibited elevated sTRAIL (30.5 ± 3.9 pg/ml, *P* = 0.035). Maternal serum levels of calprotectin were neither diagnostic nor prognostic to early pregnancy failures (*P* > 0.05).

**Conclusions:**

The study indicated of sTRAIL as a potential predictive biomarker in maternal serum for early pregnancy complications.

## 1 Introduction

Recurrent miscarriage (RM), defined as three or more consecutive pregnancy losses before 20–22 weeks pregnancy, affects 1–3% of couples aimed at childbirth [1–3]. While fetal chromosomal abnormalities represent the major factor behind sporadic miscarriages [4,5], they account for a smaller fraction of miscarriage events in RM couples [6,7]. Currently available diagnostic procedures allow to identify clinical conditions increasing their risk to pregnancy failure and to offer appropriate management options only in 50% of RM couples [1]. The known risk factors for developing of RM are maternal thrombophilic disorders or antiphospholipid syndrome (5–15% in RM vs 2–5% in control women), uterine malformations (10–25% in RM vs ∼5% in controls), maternal immunological and endocrine disturbances, parental balanced chromosomal rearrangements (3–6% of couples) [1]. Nevertheless, half of RM couples are classified as idiopathic with no identifiable cause [8]. Early recognition of a potential risk to pregnancy loss and systematic monitoring has beneficial effect in increasing live birth rates in RM couples [8,9]. The preferred medical supportive care includes determination of hCG rising serum concentrations in early pregnancy and frequent ultrasound examinations [9]. However, only a limited number of potential predictive biomarkers of threatening miscarriage have been proposed [10–13].

Successful implantation, trophoblast invasion and placental development are key processes in early pregnancy, and placental insufficiency increases a risk of miscarriage and other pregnancy complications [14,15]. Due to the difficulties in obtaining first trimester placental tissue material, only a limited number of investigations on targeted gene expression in placentas from RM cases have been conducted [16–18]. To our knowledge, no whole-genome gene expression profile has been reported for RM placental tissue. The current study aimed (i) to address the whole-genome differential gene expression in the first trimester placental tissue in cases of RM compared to gestational-age matched uncomplicated pregnancies, and (ii) to determine the protein levels of identified RM related genes in maternal serum in order to evaluate their potential as biomarkers for predicting early pregnancy complications. In RM placentas, the study detected increased expression of TNF-related apoptosis-inducing ligand (*TNFSF10*; *TRAIL*) and *S100A8* encoding for inflammatory marker calprotectin (heterodimer S100A8/A9). The study indicated sTRAIL as a potential predictive biomarker in maternal serum for early pregnancy complications.

## 2 Methods

### 2.1 Study subjects

**Fig. 1:**
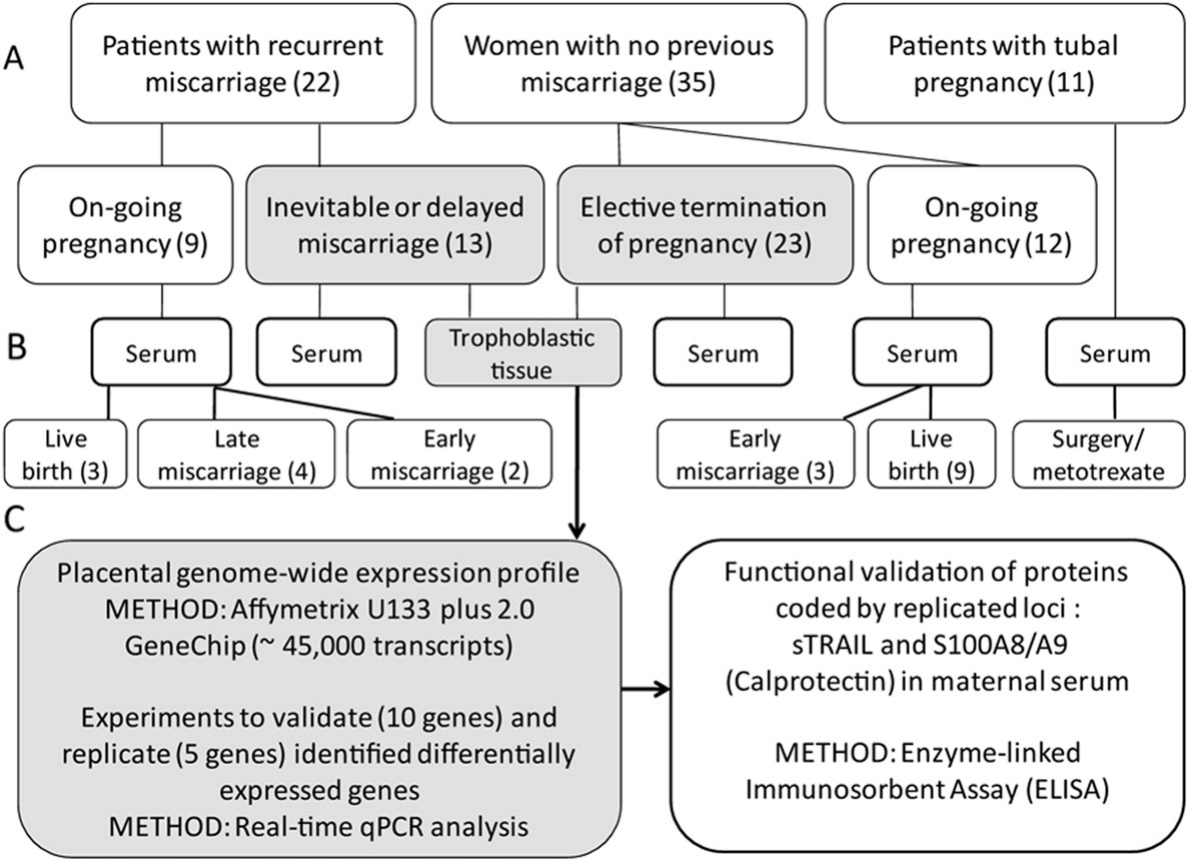
Study design. A. Patient groups analyzed in the study included patients diagnosed with recurrent miscarriage (RM), women with no miscarriages in their reproductive history as well as or patients with tubal pregnancy. RM was defined as three and more consecutive pregnancy losses. Index pregnancies at the recruitment were classified as ongoing or terminated. B. Biological material analyzed in the study included placental tissue (boxes in gray background, solid line) and maternal serum samples (unfilled boxes, dashed line). Trophoblastic tissue for genome-wide expression profiling was obtained during the surgical evacuation of uterus in patients with recurrent (≥3rd case) miscarriage or elective termination of normal uncomplicated pregnancy (ETP) before 12th gestational weeks. Serum samples were taken during an office visit at the first trimester of pregnancy. The course of the index pregnancy was followed until successful delivery or pregnancy loss. Treatment with methotrexate or laparoscopic surgery due to tubal pregnancy was applied after blood sampling. C. Experimental design included mRNA expression analysis and functional assays. Differentially expressed genes identified from genome-wide expression profile in trophoblastic tissue were confirmed and replicated by real-time RT-qPCR using locus-specific Taqman assays. Concentration of proteins sTRAIL and S100A8/A9 (calprotectin) coded by the loci exhibiting significant overexpression in RM placentas, was measured in maternal blood serum samples.

Study participants were recruited at Women's Clinic of Tartu University Hospital, Estonia during 2003–2011. The inclusion and analysis of collected human samples (placenta, blood serum) in each study stage are detailed on Fig. 1. The study was approved by the Ethics Review Committee on Human Research of the University of Tartu, Estonia (protocols no 17/9, 16.06.2003 and 180/M-15, 23.03.2009) and was conducted according to Declaration of Helsinki principles. A written informed consent to participate in the study was obtained from each individual.

#### 2.1.1 Study subjects for placental gene expression experiments

Subjects included into the analysis of differential gene expression in trophoblastic tissue comprised of women (i) with recurrent miscarriage with no identifiable cause (RM patients; age 31.8 ± 1.5 years, gestational age at the miscarriage 67.7 ± 6.6 days) and (ii) electively terminated normal first trimester pregnancies with no maternal or fetal clinical complications until the termination of the pregnancy (ETP controls; age 26.7 ± 1.4 years, gestational age 63.0 ± 2.7 days) (Fig. 1A). The recruited RM patients (*n* = 13; parity 0–2, mean 0.7; gravidity 3–8, mean 4.3) had experienced at least three consecutive miscarriage events. The ETP control group (*n* = 23; parity 0–4, mean 1.5; gravidity 2–9, mean 3.8) had no clinically confirmed miscarriages in their clinical reproductive history. The following risk factors of pregnancy loss had been excluded in RM cases: abnormal menstrual cycle (<21 days and >35 days), genital infections, antiphospholipid syndrome (positive anticardiolipin and/or β2 glycoprotein 1 antibodies), thrombophilic mutations (*Factor V* Leiden c.6191G>A, p.Arg506Gln; *Coagulation factor II* c.20210G>A) and genital tract anomalies (ultrasound examination or hystero-sonography) in RM women, and abnormal karyotype of either of the partners. Neither anticoagulant nor immunological treatment was applied during the pregnancy. Three patients had been prescribed dydrogesterone at variable gestational ages, with variable treatment period and dosage per day. Due to low number of involved patients and multiplicity of treatment schemes, the effect on mRNA expression was not assessed.

At the initiation of the project, the study subjects were randomly assigned into either the discovery (*n* = 10) or the replication sample (*n* = 26) set. Trophoblastic tissue and maternal serum were sampled from recruited patients on the day of the uterine curettage due to inevitable or delayed miscarriage (RM patients) or elective determination of the pregnancy the patient (ETP controls). Inevitable miscarriage was defined as an ongoing process of miscarriage and delayed miscarriage as the presence of clearly developed fetus without detectable heart beats at ultrasound examination [19,20].

#### 2.1.2 Study subjects for maternal serum ELISA

Enzyme-linked immunosorbent assay (ELISA) was applied for the quantification of sTRAIL and S100A8/A9 (calprotectin) proteins in maternal serum obtained from: (i) same individuals studied for in the placental gene expression analysis (13 RM cases, 23 ETP controls); (ii) patients with ongoing pregnancies recruited at the 4–13th weeks of gestation (*n* = 32) (Fig. 1). All maternal serum samples have been stored at −80 °C.

Among the 21 women with ongoing intrauterine pregnancy, 12 patients had no history of miscarriage and 9 patients had experienced RM before the index pregnancy; additionally 11 women were recruited at their diagnosis of a tubal pregnancy. Among the 12 women with no history of RM, in nine cases the index pregnancy resulted in the birth of a single live baby and in three cases the women experience early or delayed miscarriage one to two weeks after serum sampling. Among the nine prospectively monitored patients with the clinical history of RM, the index pregnancy ended with a birth of single live baby in one-third of the cases (*n* = 3). The rest of the patients (*n* = 6) experienced again loss of the index pregnancy monitored in this study. Two cases had an early miscarriage one week after recruitment. Four patients experienced late pregnancy loss between 16 and 27th gestational weeks due to placental infarct, chorioamnionitis or severe Parvovirus infection. Blood serum sampling of the 11 women with ongoing tubal pregnancy was performed a day before treatment of the patients with methotrexate or laparoscopic surgery.

### 2.2 Tissue collection and RNA extraction

Placental tissue samples were obtained during elective surgical abortion of uncomplicated pregnancy (ETP individuals) or uterine curettage due to recurrent incomplete or delayed miscarriage (RM individuals), performed in Women's Clinic of Tartu University Hospital in 2003–2006. Karyotype of the collected placental samples was unavailable. Placental samples were immediately washed with 0.9% saline solution to remove maternal blood, snap-frozen in liquid nitrogen and stored at −80 °C or placed into RNAlater solution (Ambion, Austin TX, USA) and kept at −20 °C for RNA analysis. RNA was extracted from 200 mg of homogenized placental tissue (containing trophoblastic and decidual material) of each subject using TRIzol^®^ Reagent (Invitrogen, Paisley, UK) according to supplier's recommendations. To remove the residual phenol from TRIzol^®^-based extraction, 100 μl of extracted total RNA was further cleaned with NucleoSpin^®^ RNA II mini spin columns (Macherey–Nagel, Düren, Germany) following the manufacturer's protocol. Purity level and concentration was measured using NanoDrop 1000 Spectrophotometer (Thermo Fisher Scientific, Waltham, MA, USA). Aliquots of the RNA used for microarray analysis were evaluated by the Agilent RNA 6000 Nano LabChip kit on an Agilent Bioanalyzer 2100 system (Agilent Technologies, Santa Clara, CA, USA).

### 2.3 Microarray hybridization

Differential gene expression in placental tissues in RM (*n* = 4) compared to normal first trimester uncomplicated pregnancies (*n* = 6) was addressed using Affymetrix Human Genome U133 plus 2.0 GeneChip according to manufacturer's recommendations (Affymetrix, Santa Clara, CA, USA). In brief, 1 μg of purified total RNA was used to generate the first strand cDNAs using SuperScript™ II Reverse Transcriptase (Invitrogen) and T7-oligo (dT) primer. Next, *in vitro* transcription reaction for cRNA amplification and biotin labeling was performed in the presence of T7 RNA Polymerase and a biotinylated nucleotide analog/ribonucleotide mix. Biotin-labeled cRNA targets were cleaned up, fragmented, and hybridized to GeneChip expression arrays.

#### 2.3.1 Microarray data analysis

The microarray data generated in the study is Minimum Information About a Microarray Experiment (MIAME) compliant and the raw data has been deposited to a MIAME compliant database the Gene Expression Omnibus (GEO) data repository (accession no GSE22490). Quality control (QC) was performed by *affy* package of Bioconductor, an open-source software project based on R [21]. QC criteria included comparison of average intensity, correlation with median intensity of other GeneChips, *GAPDH* 3′→5′ and *β-actin* 3′→5′, scaling factor, percentage of presence calls, average background, intensities of positive and negative border elements (Fig. S1). GeneChip cel-files were imported to dChip [22] (http://biosun1.harvard.edu/complab/dchip/) and after QC analyzed using perfect match (PM)/mismatch (MM) modeling and invariant set normalization as previously described [23,24]. We filtered for the genes with high signal intensity (dChip signal threshold = 100). Sample comparison (ETP against RM) was done with *t*-test, and fold-changes were calculated to evaluate differential expression of genes. Estimation of the empirical False Discovery Rate (FDR) [25] is described in Supplemental Data, Text S1. Hierarchical clustering of probe sets was performed by centroid linkage and the distance between two genes was defined as 1 − *r*. Allocation of identified genes to biological processes was performed using Gene Ontology Annotation Database (http://www.ebi.ac.uk/GOA/).

### 2.4 Reverse transcription quantitative PCR (RT-qPCR) and gene expression data analysis

One μg of total RNA was reverse transcribed to cDNA using SuperScript™ III First-Strand Synthesis SuperMix for RT-qPCR kit (Invitrogen) in accordance with manufacturer's instructions. Quantitative gene expression analyses were performed with commercially available pre-made TaqMan Gene Expression Assays (Table S1; Applied Biosystems, Carlsbad, CA, USA) using a duplex qPCR of target sequence and endogenous control (*HPRT*, Applied Biosystems). The qPCR reactions (95 °C for 15 min; 40 cycles of 15 s at 95 °C and 1 min at 60 °C) were performed in triplicate with ABI 7900HT Real-time PCR system (Applied Biosystems) using ABsolute™ QPCR ROX Mix (Thermo Fisher Scientific) or HOT FIREPol^®^ Probe qPCR Mix (Solis BioDyne, Tartu, Estonia). Negative controls contained either non-reverse transcribed RNA or lacked template inputs.

Relative quantification of RT-qPCR data was determined by comparative *C*_*T*_ method, mean value of normalized expression was calculated by averaging three independently calculated normalized expression values of the triplicate (Equation No 2 in [26]). The calculations and efficiency corrections were carried out with qGENE software [27]. The median expression level of the control group was selected as a calibrator. All statistical analyses were performed by R 2.8.1, a free software environment for statistical computing and graphics (http://www.r-project.org/). Logistic regression model adjusted by maternal and gestational age was applied to assess differential gene expression between RM cases and controls quantified by RT-qPCR. The stringent Bonferroni significance threshold for multiple testing correction was calculated *α* = 0.05/*N* = 0.005, where *N* = 10 represents the number of independent Taqman RT-qPCR gene-probes.

### 2.5 ELISA analysis of sTRAIL and S100A8/S100A9 heterocomplex in maternal serum

Enzyme-linked immunosorbent assay (ELISA) was used to assess the concentration of sTRAIL (DuoSet ELISA development kit #DY375, lot #1240906; R&D Systems Europe, Ltd., Abingdon, UK) and S100A8/A9 (MRP8/14 ELISA kit #S-1011, lot #14E-1101; BMA Biomedicals, Augst, Switzerland) proteins in maternal serum. ELISA assays were conducted following manufacturer's instructions, details are provided in Supplemental Data, Text S2. The raw data were analyzed by curve fitting ReaderFit software with 5-parameter logistic model (www.readerfit.com). All statistical analyses were performed by R 2.13.1. Logistic regression models were applied to assess the differences between the study groups in the concentration of sTRAIL and S100A8/A9 in maternal serum. The analyses were adjusted for gestational and maternal age. Natural log-transformation was applied to all quantitative data to improve the approximation of normal distribution. Correlation between the relative mRNA expression (RT-qPCR) in placenta and protein level in maternal serum was assessed with Pearson correlation test (coefficient *r*). *P* ≤ 0.05 was considered as statistically significant.

## 3 Results

### 3.1 Genome-wide differential gene expression in RM placentas

Whole-genome differential gene expression patterns in placental tissue obtained from four recurrent miscarriage (RM) and six uncomplicated first trimester pregnancies were addressed using GeneChip (Affymetrix) expression arrays (Fig. 1). In total, 27 differentially expressed genes (representing 30 transcripts with nominal *P*-values < 0.05) in RM compared to control placentas were identified, 12 genes with increased and 15 loci with reduced transcript levels (Table 1, Fig. S3). However, none of the *P*-values remained significant after correction for multiple testing with FDR and thus, GeneChip profiling was considered as exploratory analysis for hypothesis generation. For the further confirmatory experiments of the discovery observations, genes were selected based on their fold-changes. In GeneChip dataset, alternations in gene expression in RM placentas ranged from ∼2.39 fold decrease in *ASMTL* mRNA level (*P*-value 0.048) to more than 4.11 fold increased expression of *S100A8* transcripts (*P*-value 0.040). The expression level of *S100A9*, the dimerization partner of *S100A8* in the assembly of heterocomplex S100A8/A9 protein (calprotectin) was also up-regulated (fold-change 1.81; *P*-value 0.040). It is noteworthy, that three probes targeting the *TRAIL* (*TNFSF10*) gene were among the six transcripts with the highest fold-change (fold-changes 2.16–2.75; *P*-value ≤0.032). The ‘Volcano’ plot positioned *TRAIL* (*TNFSF10*) among the genes exhibiting the highest differential expression (top fold-change) in RM combined with the best statistical support (lowest *P*-values) (Fig. S2). Among the detected 27 differentially expressed genes in RM placentas, an enrichment of genes involved in cell communication and signaling (*CALR*, *CCR1*, *LYN*, *NENF*, *PTN*, *S100A9*, *TRAIL*), inflammatory and immune response (*CD163*, *CCR1*, *SECTM1*, *S100A8*, *S100A9*, *TRAIL*) was observed (Table S2).

### 3.2 Significantly increased levels of *S100A8* and *TRAIL* transcripts in RM placentas

Although GeneChip profiling had failed to identify any transcripts exhibiting statistically significant (after FDR) evidence for differential expression in RM placentas, it provided a list of genes with the highest fold-changes, and thus with potential importance. Top genes in this list were subjected to further experimental confirmation by an independent method for mRNA quantification, and by analyzing an independent sample set. First, ten genes (*ASMTL*, *BRD1*, *CALR*, *CCR1*, *CD163*, *NENF*, *PTN*, *S100A8*, *SNAI2*, *TRAIL*) showing a fold-change greater than two (up- and down-regulated) in RM placental material were subjected to experimental confirmation by RT-qPCR using identical samples as analyzed at the expression microarray (Table 2). One gene, *SECTM*, was excluded from RT-qPCR due to incompatibility with the pre-made Taqman assay. mRNA quantification by Taqman confirmed significantly altered expression in RM placental tissue for five genes (logistic regression: *S100A8*, *P* = 2.4 × 10^−4^, fold-change 4.98; *TRAIL*, *P* = 2.7 × 10^−3^, fold-change 2.46; *CD163*, *P* = 2.5 × 10^−2^, fold-change 2.61; *CCR1*, *P* = 2.0 × 10^−2^, fold-change 1.79; *SNAI2*, *P* = 2.0 × 10^−3^, fold-change – 1.90).

**Fig. 2:**
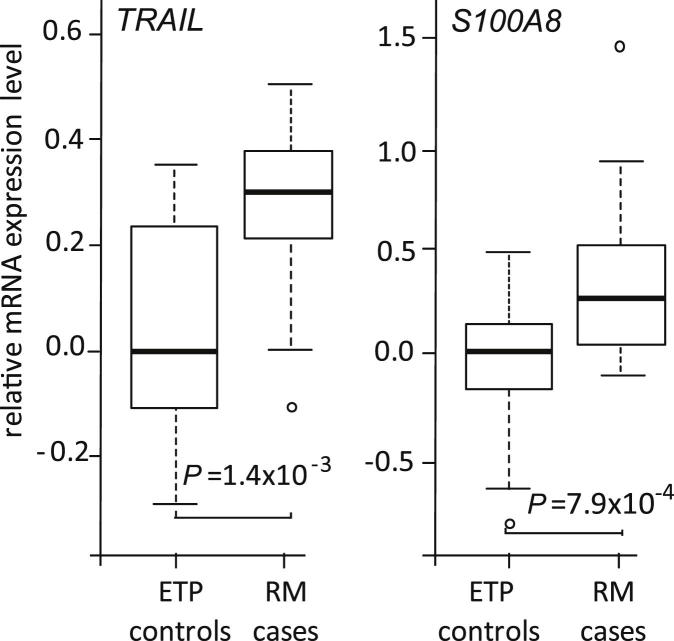
Significantly increased expression of *TRAIL* and *S100A8* in placental tissue from recurrent miscarriage cases. TaqMan primer/probe sets were applied for quantification of gene expression of studied using total RNA isolated from placental tissue of recurrent miscarriage (RM) and electively terminated uncomplicated pregnancies (ETP) in discovery (RM cases, *n* = 4; ETP controls, *n* = 6) and in replication (RM cases, *n* = 9; ETP controls, *n* = 17) sample-sets. Samples of total RNA isolated from placentas of recurrent miscarriage (RM cases; *n* = 13) and electively terminated uncomplicated pregnancies (ETP controls, *n* = 23) were analyzed using TaqMan primer/probe sets. Presented boxplots summarize the distribution of relative mRNA expression in the joint sample set of the discovery and the replication specimen. The median expression level of the ETP group was selected as calibrator and relative mRNA expression levels are shown on logarithmic scale. *P*-values for the comparison of gene expression between RM and ETP groups were estimated by logistic regression model with gestational age and maternal age as cofactors.

Next, differential expression of the five genes confirmed by RT-qPCR was further analyzed in an independent replication sample (*n* = 26; 9 RM placentas; *n* = 17 controls). Significantly increased expression of *S100A8* (*P* = 3.3 × 10^−2^, fold-change 1.97) and *TRAIL* (*P* = 4.8 × 10^−2^, fold-change 1.45) in RM placentas (Table 2; Fig. S3) was replicated. Combined analysis of the discovery and the replication RT-qPCR datasets enhanced the statistical support for the increased transcript levels of *S100A8* (*P* = 7.9 × 10^−4^, fold-change 2.56) and *TRAIL* (*P* = 1.4 × 10^−3^, fold-change 1.68) in placentas from RM cases (Fig. 2; Table 2). For both genes, differential expression in RM compared to control placentae was observed in very early gestation at the 4–7 weeks (*S100A8*: fold-change 6.91, *TRAIL*: fold-change 4.02) as well as in the second half of first trimester at the 8–13 gestational weeks (*S100A8*: fold-change 1.33, *TRAIL*: fold-change 2.00).

### 3.3 Significantly elevated sTRAIL in maternal serum in early pregnancy complications

ELISA assay was applied to estimate the concentration of the soluble form of TNF-related apoptosis-inducing ligand (sTRAIL) in maternal serum in normal first trimester gestation and in cases of failed pregnancies. The maternal serum of the failed pregnancy cases had been collected either at the miscarriage event, or prospectively during an ongoing gestation with no prognostic information on its possible outcome (2–50 days prior occurrence of unpredicted pregnancy loss). In addition, sTRAIL concentration was determined in sera of patients diagnosed of tubal pregnancy, the second most common early pregnancy complication.

**Fig. 3:**
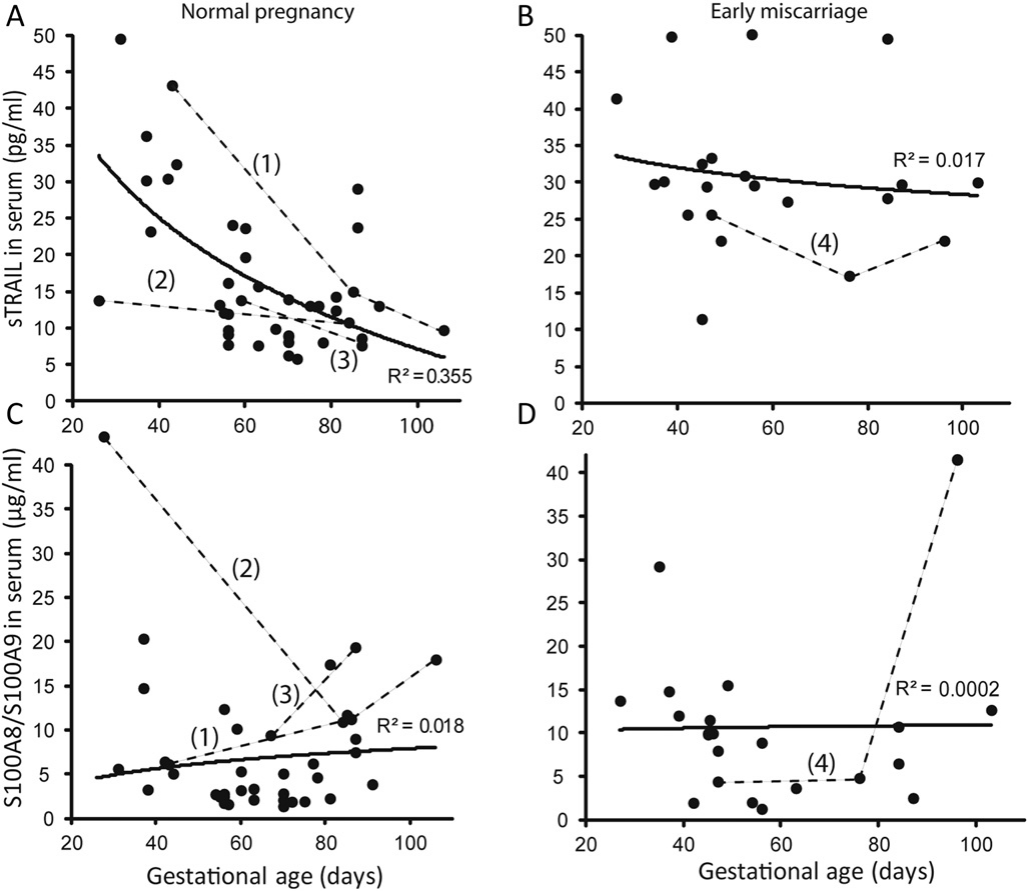
Dynamics of sTRAIL (A,B) and heterocomplex S100A8/S100A9 (C,D) level in first trimester maternal serum in normal and miscarried pregnancies. Normal pregnancy (a, c; *n* = 35) group included serum samples collected at the first trimester of gestation (gestational age shown in days) from individuals whose pregnancy ended with a live birth of a singleton baby or from women's who had decided for elective termination of an uncomplicated pregnancy (ETP group). Early miscarriage group (b, d; *n* = 18) included maternal sera sampled either at the time of inevitable or delayed miscarriage (RM group) or prospectively during the ongoing index pregnancy, which spontaneously terminated with a miscarriage before 13th gestational weeks. A logarithmic trendline describes the dynamics of sTRAIL and S100A8/S100A9 (calprotectin) concentration in serum during the pregnancy and *R*^2^ estimate equals the square of the correlation coefficient between the observed and modeled (predicted) data values. Serial serum measurements during the first trimester from four individuals, numbered (1) to (4) are shown with dashed line.

In the first trimester normal pregnancy the serum concentration of sTRAIL exhibited its peak concentration at the 4–7th gestational week (mean 32.4 pg/ml, median 32.6 pg/ml, range 13.7–49.5 pg/ml; Fig. 3A) followed by a gradual decrease toward the end of the first trimester (mean level during the 8–13th gestational week 12.7 pg/ml, median 12.0 pg/ml, range 5.8–29.0 pg/ml). In contrast, no similar decrease in the maternal serum sTRAIL concentration was detected in pregnancies that ended with early miscarriage (Fig. 3B). In cases of pregnancy loss the circulating protein level during the 8–13th gestational week remained as high as at the beginning of the pregnancy (mean 32.5 pg/ml, median 29.8 pg/ml, range 17.3–50.1 pg/ml).

**Fig. 4:**
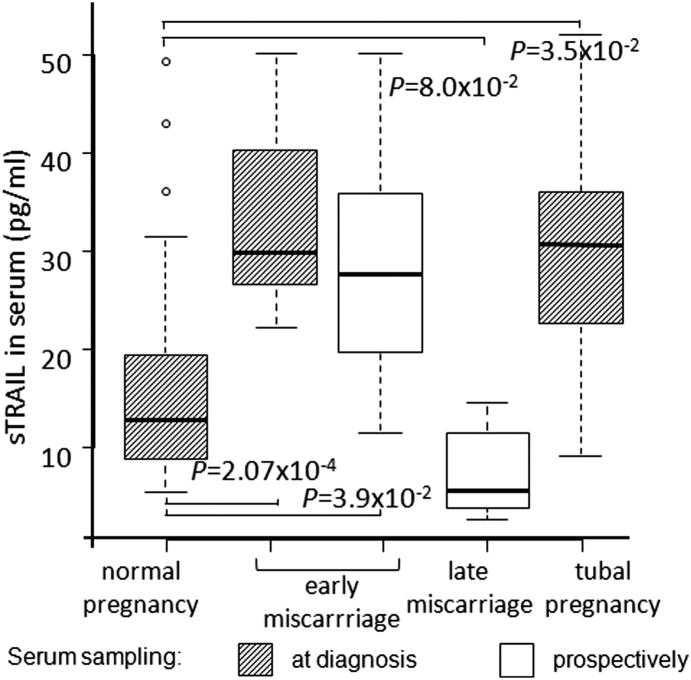
Concentration of sTRAIL in first trimester maternal serum in normal and failed pregnancies. Normal pregnancy (*n* = 35) included serum samples collected at the first trimester of gestation from individuals whose pregnancy ended with a live birth or from women's who had decided for elective termination of an uncomplicated pregnancy (ETP group). Maternal serum in early miscarriage group had been sampled either at the time of inevitable or delayed miscarriage (after clinical appearance, *n* = 13) or prospectively during the ongoing index pregnancy, which spontaneously terminated with a miscarriage before 13th gestational weeks (*n* = 5). Serum had been sampled 2–50 days before clinical appearance of pregnancy loss. Late miscarriage group (*n* = 4) was defined as a miscarriage after 12th gestational weeks (16 and 20 weeks) or as extremely preterm (24 and 27 weeks) delivery of a stillbirth baby; the analyzed maternal serum had been sampled prospectively in the first trimester of pregnancy. The serum from the patients with tubal pregnancy (*n* = 11) was taken before application of surgical or medical treatment. The boxes represent the 25 and 75th percentiles. The median is denoted as the line that bisects the boxes. The whiskers are lines extending from each end of the box covering the extent of the data on 1.5 *X* interquartile ranges. Circles represent the outlier values. *P*-values reflecting the differences between groups were estimated by logistic regression and adjusted for maternal and gestational age.

When compared to normal first trimester pregnancy (sTRAIL 16.1 ± 1.6 pg/ml, median 13.0 pg/ml), significantly higher maternal serum concentration of sTRAIL was detected both (i) at the recurrent miscarriage event (33.6 ± 4.3 pg/ml, median 29.7 pg/ml; *P* = 0.00027; Fig. 4), as well as (ii) in uncomplicated gestation at the time of serum sampling, which terminated with an unexpected first trimester pregnancy loss (28.5 ± 4.4 pg/ml, median 27.7 pg/ml; adjusted *P* = 0.039). In addition, women with diagnosed tubal pregnancy also exhibited significantly elevated concentration of sTRAIL in their circulation (30.5 ± 3.9 pg/ml, median 31.9 pg/ml; adjusted *P* = 0.035). In contrast to the cases with early pregnancy failure, sTRAIL concentrations measured in women with an uncomplicated pregnancy at the time of serum sampling, but developing a late miscarriage or extremely preterm delivery of a stillbirth baby (16–27 weeks of gestational age) did not differ statistically from the sTRAIL serum levels in normal pregnancy resulting in the birth of a healthy child (mean 7.6 ± 2.3 pg/ml, median 5.6 pg/ml; adjusted *P* > 0.05).

In the cases with data for both placental mRNA expression of *TRAIL* and maternal serum concentration of sTRAIL, the two measurements were significantly correlated (Pearson's correlation coefficient *r* = 0.60, *P* = 0.002).

### 3.4 Maternal serum level of S100A8/A9 is not indicative to early pregnancy failure

No temporal dynamics during the first trimester of normal and failed pregnancies was detected in the maternal serum levels of heterodimeric complex of S100A8/A9 (calprotectin) by ELISA assays (Fig. 3C–D). Maternal serum levels of S100A8/A9 were neither diagnostic (sampled at the occurred miscarriage or tubal pregnancy, *P* > 0.05) nor prognostic (sampled prospectively in patients, who later experienced pregnancy loss, *P* > 0.05) to pregnancy failures, when compared to normal pregnancy at the same gestational age. In the cases with the available data for placental mRNA expression of *S100A8* and maternal serum concentration of S100A8/A9, the two measurements were not correlated (*r* = 0.04, *P* = 0.8).

## 4 Discussion

The current study represents the first genome-wide expression profiling of placental tissues from recurrent miscarriage (RM) compared to uncomplicated first trimester pregnancy. The identified 27 differentially expressed genes (nominal *P* < 0.05) act in the pathways involved in cell signaling and apoptosis, inflammation and environmental response (Table S2). The main limitation of the discovery study was small sample resulting in limited statistical power in whole-genome expression profiling. Additionally, possible inclusion of some aneuploid miscarriages may have weakened the analysis, as the karyotype of miscarried embryos was unavailable. The limitations of the GeneChip exploratory analysis were overcome by two rounds of confirmatory experiments using quantitative methods (RT-qPCR and serum measurements), which targeted individual loci. Significantly increased mRNA expression of apoptosis-inducing ligand *TRAIL* and inflammatory marker *S100A8* was confirmed (Fig. 2; Table 2). Measurement of the soluble forms of coded proteins in maternal serum in the first trimester gestation revealed sTRAIL as a potential novel non-invasive predictive biomarker for pregnancy outcome (Figs. 3 and 4). Our pilot dataset suggests that the level of maternal serum sTRAIL is predictive of the potential risk of pregnancy failure from the 8th gestational week. It is noteworthy that a recent study has independently demonstrated significantly elevated levels of sTRAIL immediately after miscarriage in RM patients compared to that in first-trimester normal pregnant women [13].

Apoptosis plays an important role in the normal development of human placenta and an altered balance between proliferation and apoptosis of villous trophoblast is associated with abnormal pregnancies [28,29]. Increased levels of villous trophoblast apoptosis have been identified in several placental pathologies including early pregnancy loss, tubal pregnancy, preeclampsia, intrauterine growth restriction and gestational trophoblastic disease [17,28–32]. *TRAIL* encodes for TNF-related apoptosis-inducing ligand that is expressed on the surface of immune cells in many tissues. It is a type II transmembrane protein and can be cleaved by a cysteine protease to generate a soluble form sTRAIL [33]. In the villous placenta *TRAIL* is expressed constantly throughout gestation at the protein level [34]. The expression of *TRAIL* in trophoblast cell culture and release of sTRAIL into culture media was reported to decrease concordantly during differentiation and syncytialisation processes [35]. In the present study a gradual decrease of maternal sTRAIL during the first trimester of normal pregnancy was documented. Interestingly, *TRAIL* and its receptors appear to be differentially expressed in villous placenta. Expression of *TRAIL* and its two decoy receptors DcR1 and DcR2 is localized predominantly in the syncytiotrophoblast, whereas cytotrophoblast cells have been shown to express high levels of DR4 and DR5 [34,36] that trigger apoptotic signaling via activation of the classic caspase-dependent ‘death’ pathway [37]. In contrast, DcR1/DcR2 and a soluble *TRAIL* receptor osteoprotegerin antagonize apoptotic signaling and may protect resident cells of the fetal membranes against the pro-apoptotic effects of *TRAIL* during pregnancy [35,38]. Additionally, the trophoblast might use *TRAIL* as a non-apoptotic signal influencing the cytokine milieu of the maternal endometrium [39]. The presence of *TRAIL* has been also shown at the tubal implantation site, possibly contributing to trophoblast invasion in cases of extrauterine pregnancy [40]. Taken together, there is an important balance between cell death and survival signaling upon *TRAIL* binding [41]. The increased level of sTRAIL may reflect either already initiated process of fetal rejection or indicate to abnormal development and syncytialization of cytotrophoblast cells.

In addition to *TRAIL*, the current study detected significantly elevated placental mRNA expression of *S100A8* in cases of RM. Heterodimeric S100A8/A9 protein (calprotectin) has antimicrobial, cytostatic, antiproliferative, apoptosis-inducing and chemotactic properties and is involved in many physiological and pathological processes in female reproductive tract [42]. The significantly increased concentrations of S100A8/A9 in amniotic fluid or in maternal serum have been reported in intra-amniotic inflammation, preterm labor and preeclampsia [42–44]. However, in the present study the increased placental mRNA expression of *S100A8* and *S100A9* in RM placentas was not reflected in the level of calprotectin in maternal serum and may rather reflect a local inflammation process of the tissues at the maternal–fetal interphase during the rejection process of the pregnancy.

In summary, we showed *TRAIL* and *S100A8* as differentially expressed genes in placental tissue from recurrently miscarried compared to uncomplicated pregnancies. Significantly increased level of sTRAIL was measured in maternal serum during the first trimester of pregnancy with unfavorable endpoint, exhibiting potential as a predictive non-invasive biomarker for early pregnancy complications.

## Figures and Tables

**Table 1:** Placental gene expression patterns of recurrent miscarriage (RM; *n* = 4) and electively terminated uncomplicated pregnancies (ETP; *n* = 6) were assessed using Affymetrix HG-U133 plus 2.0 GeneChips.

Gene (abbreviation)	Fold-change	90%CI	Nominal *P*-value
*Transcripts with increased expression in RM placenta*			
S100 calcium binding protein A8 (*S100A8*)	4.11^a^	2.13; 10.05	0.03973
CD163 molecule (*CD163*)	2.94^a^	1.56; 7.19	0.04662
Tumor necrosis factor (ligand) superfamily, member 10 (*TNFSF1* or *TRAIL*)	2.75^a^	1.93; 3.82	0.02344
Tumor necrosis factor (ligand) superfamily, member 10 (*TNFSF1* or *TRAIL*)	2.57^a^	1.79; 3.69	0.02016
Secreted and transmembrane 1 (*SECTM*)	2.38	1.60; 3.61	0.02776
Tumor necrosis factor (ligand) superfamily, member 10 (*TNFSF1* or *TRAIL*)	2.16^a^	1.49; 3.14	0.03204
Bromodomain containing 1 (*BRD1*)	2.12^a^	1.25; 5.25	0.03722
Chemokine (C–C motif) receptor 1 (*CCR1*)	2.03^a^	1.50; 2.69	0.02940
Potassium voltage-gated channel, Isk-related family, member 3 (*KCNH2*)	1.97	1.35; 2.94	0.03691
Bromodomain containing 1 (*BRD1*)	1.92^a^	1.34; 3.11	0.01233
S100 calcium binding protein A9 (*S100A9*)	1.81	1.24; 3.25	0.02793
Branched chain aminotransferase 1 (*BCAT1*)	1.63	1.32; 2.10	0.00362
RAB11 family interacting protein 1 (*RAB11FIP1*)	1.52	1.26; 1.87	0.00502
CCAAT/enhancer binding protein (C/EBP), delta (*CEBPD*)	1.5	1.21; 1.84	0.02857
v-yes-1 Yamaguchi sarcoma viral related oncogene homolog (*LYN*)	1.39	1.23; 1.59	0.00167
*Transcripts with decreased expression in RM placenta*			
Acetylserotonin O-methyltransferase-like (*ASMTL*)	−2.39^a^	−1.47; −3.51	0.04775
Calreticulin (*CALR*)	−2.26^a^	−1.64; −3.15	0.00636
Pleiotrophin (*PTN*)	−2.23^a^	−1.36; −4.72	0.02488
Neuron derived neurotrophic factor (*NENF*)	−2.2^a^	−1.45; −3.74	0.01409
Snail homolog 2 (Drosophila) (*SNAI2*)	-2^a^	−1.40; −2.76	0.02495
Thymidine kinase, soluble (*TK1*)	−1.88	−1.26; −3.01	0.03030
HOXA distal transcript antisense RNA (non-protein coding) (*HOTTIP*)	−1.86	−1.42; −2.40	0.01283
C1GALT1-specific chaperone 1 (*C1GALT1C1*)	−1.83	−1.29; −2.60	0.02606
F-box protein 22 (*FBXO22*)	−1.83	−1.33; −2.57	0.01609
Collagen, type V, alpha 1 (*COL5A1*)	−1.67	−1.31; −2.12	0.01081
Eukaryotic translation initiation factor 2B, subunit 2 beta (*EIF2B2*)	−1.58	−1.22; −2.00	0.03363
Low density lipoprotein receptor (*LDLR*)	−1.58	−1.28; −2.03	0.01001
Calumenin (*CALU*)	−1.55	−1.25; −1.96	0.01028
Synaptophysin-like 1 (*SYPL1*)	−1.5	−1.20; −1.89	0.01865
Mitochondrial ribosomal protein L19 (*MRPL19*)	−1.4	−1.21; −1.62	0.00821

The normalized log intensity values for the 30 most highly up-regulated and down-regulated differentially expressed probe sets (nominal *P-*value < 0.05; fold-change difference > 1.2).

a Selected for confirmation with RT-qPCR method.

**Table 2:** RT-qPCR experimental confirmation of top differentially expressed genes^a^ in Gene-chip profiling profiling in placentas from recurrent miscarriage.

Gene^a^	Identical discovery samples as used in GeneChip analysis		Independent replication samples		Joint analysis of discovery and replication samples	
	(Cases *n* = 4, controls *n* = 6)		(Cases *n* = 9, controls *n* = 17)		(Cases *n* = 13; controls *n* = 23)	
	Fold-change	*P*-value	Fold-change	*P*-value	Fold-change	*P*-value
*ASMTL*	−1.11	0.37	nd	nd	nd	nd
*BRD1*	−1.02	0.90	nd	nd	nd	nd
*CALR*	1.14	0.41	nd	nd	nd	nd
*CCR1*	1.79	0.020	−1.03	0.83	1.15	0.70
*CD163*	2.61	0.025	1.04	0.98	1.37	0.20
*NENF*	1.15	0.29	nd	nd	nd	nd
*PTN*	−1.09	0.79	nd	nd	nd	nd
***S100A8***	**4.98**	0.00024*	**1.97**	**0.033**	**2.56**	0.00079*
*SNAI2*	−1.90	0.0020*	−1.23	0.32	−1.37	0.068
***TRAIL***	**2.46**	0.0027*	**1.45**	**0.048**	**1.68**	0.0014*

a Genes with showing a fold-change greater than two (up- and down-regulated) in the GeneChip analysis in placental material from recurrent miscarriage (RM) compared to controls (Table 1). Fold-change was calculated as the difference of mean relative expression values of patient and control groups. Logistic regression model adjusted by gestational age and maternal age was applied to assess differential gene expression between RM cases and controls quantified by RT-qPCR. Statistically significant (*P* < 0.05) tests after Bonferroni correction for multiple testing are indicated in bold and with asterisk (*). nd: not done.
