# Supplementary material for: Increased placental expression and maternal serum levels of apoptosis-inducing TRAIL in recurrent miscarriage
Source: Placenta. 2013 Feb;34(2):141–8. doi: 10.1016/j.placenta.2012.11.032 (PMC3562443; doi:10.1016/j.placenta.2012.11.032)
Supplement: Supplementary file 1 [file mmc1.docx]

**LIST AND Legends of SUPPLEMENTAL DATA files**

**Text S1** Estimation of False Discovery Rate (FDR) in dChip.

**Text S2** Experimental details of Enzyme-linked immunosorbent assay (ELISA) measurements of sTRAIL and S100A8/A9 (calprotectin) in maternal serum.

**Table S1** (separate .pdf file)

Probes targeting analyzed genes in Affymetrix Human Genome U133 plus 2.0 GeneChip and gene-specific commercially available pre-made TaqMan Gene Expression Assays (Applied Biosystems, Carlsbad, CA, USA) used real-time RT-PCR experiments.

**Table S2** (separate .pdf file)

Information on the genes identified using whole-genome gene expression arrays (GeneChip, Affymetrix) as differentially expressed in placental tissue from recurrent miscarriages compared to normal pregnancy.

**Figure S1** (separate file) QC analysis of GeneChip gene expression microarray (Affymetrix Human Genome U133 plus 2.0) data by exploring hybridization signal distribution.

Signal (log) intensities in all arrays are illustrated by (A) boxplots of PM (perfect mismatch) intensities, and (B) plots of kernel density estimates of these intensities. The quality control (QC) included comparison of average intensity, correlation with median intensity of other GeneChips, *GAPDH* 3’->5’ and *β-actin* 3’->5’, scaling factor, percentage of presence calls, average background, and intensities of positive and negative border elements. For two specimens among the 12 initially assessed samples, the gene array data represented a clear outlier profile with a weak average signal (log2 intensity = 5) compared to the rest of the tested arrays (log2 intensity=7). Their signal intensity was significantly lower, scale factor was above the threshold (3-fold mean for all chips), border elements had reduced intensities and the correlation with other arrays was lower. The outlier arrays were excluded from the statistical analysis and the respective samples were not used in further experiments.

**Figure S2** (separate file). Volcano plot applied for the best-ranked probesets from microarray gene expression experiments.

Volcano plot illustrates differential expression profile between the recurrent miscarriage (RM; n=4) and electively terminated uncomplicated pregnancy (ETP; n=6) groups by highlighting in the first (horizontal) dimension 100 probesets with highest absolute fold change difference (blue diamonds), and in the second (vertical) axis 100 probesets with the lowest *P*-values (red circles; t-test). The first axis indicates biological impact of the change; the second indicates the statistical evidence, or reliability of the change. Probesets were plotted by their *P*-value (lodp) and by their log-scaled fold changes differences (d). Overlapping blue diamonds and red circles denote probesets (e.g. *TRAIL or TNFSF10*) with the highest absolute differential expression and the lowest *P*-values.

**Figure S3** (separate file). Heat map of differentially expressed genes in placentas from recurrent miscarriage patients.

Placental gene expression patterns of recurrent miscarriage (RM; n=4) and electively terminated uncomplicated pregnancies (ETP; n=6) were assessed using Affymetrix HG-U133 plus 2.0 GeneChips.

The normalized log intensity values for the 30 most highly up-regulated (*upper panel*) and down-regulated (*lower panel*) differentially expressed probe sets (nominal *P-*value < 0.05; fold-change difference > 1.2) were centered to the standardized mean values of each probe set and coloured on a range of -3 to +3. *Red* denotes up-regulated, *white* denotes intermediate, and *blue* denotes down-regulated expression levels as compared with the mean value. *Columns* contain data from a single specimen, and *rows* correspond to a single probe set. Samples are arranged from left to right and grouped as RM and ETP placentas. *Rows* are ranked by fold change values, for each probe

the abbreviation of annotated gene is provided. Clustering of samples and generation of heatmaps was performed with dChip software (Li and Wong, 2001).

## Figure S4 (separate file) RT-qPCR confirmation from (A) replication samples for the five validated differentially expressed genes (*CCR1, CD163, S100A8, SNAI2, TRAIL*); (B) joint discovery and replication samples for three non-replicated loci (*CCR1, CD163, SNAI2)*.

## TaqMan primer/probe sets were applied for quantification of gene expression of studied using total RNA isolated from placental tissue of recurrent miscarriage (RM) and electively terminated uncomplicated pregnancies (ETP) in discovery (RM cases, n=4; ETP controls, n=6) and in replication (RM cases, n=9; ETP controls, n=17) sample-sets. The replication specimen had no overlap with the discovery sample use for microarray expression profiling. Presented box-plots summarize the distribution of relative mRNA expression. The median expression level of the ETP group was selected as calibrator and relative mRNA expression levels are shown on logarithmic scale. *P*-values were estimated by logistic regression.

**Text S1** **Estimation of False Discovery Rate (FDR) in dChip.**

In dChip (1), the empirical False Discovery Rate (FDR) (2) for multiple testing adjustment is estimated by permutation. The original comparison criteria are applied to the sample-wise permuted datasets and the number of obtained genes at each such permutation is recorded. After a number of permutations, we get values of the number of genes in the obtained gene lists. The median of these values is reported as the median FDR, and the 90-th percentile of these values is reported as 90% FDR, a more conservative FDR estimate.

References:

# Li C, Wong WH 2001 Model-based analysis of oligonucleotide arrays: expression index computation and outlier detection. Proc Natl Acad Sci U S A 98:31-36

# Storey JD, Tibshirani R 2003 Statistical significance for genomewide studies. Proc Natl Acad Sci U S A 100:9440-9445

**Text S2 Experimental details of Enzyme-linked immunosorbent assay (ELISA) measurements of sTRAIL and S100A8/A9 (calprotectin) in maternal serum**

ELISA assays were conducted according to the manufacturer’s instructions and all the measurements were performed at least in duplicate.

**sTRAIL:** For measurement of sTRAIL protein expression an ELISA kit (DuoSet ELISA development kit #DY375 (lot #1240906); R&D Systems Europe, Ltd., Abingdon, UK) was used according to the manufacturer’s instructions. In brief, high-binding, flat-bottom polypropylene microplates (Costar^®^, R&D Systems) were coated overnight at room temperature with 100 μL of mouse anti-human TRAIL antibody (2.0 μg/ml). The plate was washed three times with PBS containing 0.05% Tween-20 (PBST) and blocked with Reagent Diluent (PBS containing 1% bovine serum albumin, R&D Systems) for 1 hr. Either 100 μl of a sample or 100 μl of a diluted sTRAIL standard (23.5–1500 pg/ml; seven dilutions) were added to antigen-coated wells in duplicate. After 2 hours of incubation at room temperature and three washes with PBST, the plate was treated with a second biotinylated goat anti-human sTRAIL detection antibody (50 ng/ml in Reagent Diluent containing 2% heat inactivated normal goat serum) for 2 hours and after washes followed by a 1:175 dilution of Streptavidin-horseradish-peroxidase conjugate for 20 min. 100 μl of tetramethylbenzidine substrate solution (Sigma-Aldrich, Inc, Saint Louis, MO) was added for 20 min and the reaction was stopped by addition of 50 μl of Stop Reagent (Sigma-Aldrich, Inc). The absorbance at 450 nm with reference wavelength at 540 was determined for each well by using Tecan Sunrise microplate reader with Magellan™ software (Tecan Austria GmbH).

**S100A8/A9:** S100A8/A9 level in serum was measured using MRP8/14 (migration inhibitory factor-related protein) ELISA Kit (Calprotectin ELISA kit #S-1011, lot #14E-1101; BMA Biomedicals, Augst, Switzerland). Precoated and stabilized microtiter plates were coated overnight at + 4⁰ C with 100 μl calprotectin standard (10–200 ng/ml) or 100 μl diluted serum (1:100 for serums sampled more than a year ago, 1:300 for serums sampled within a year of the analysis) in assay buffer and 100 μl of peroxidate conjugated detection reagent in dilution 1:400. The plate was washed six times with PBS and immediately added 200 μl of substrate solution (tetramethylbenzidine –H_2_O_2_ and potassium chloride 1:20). After 8 min of incubation at room temperature the reaction was stopped by addition of 100 μl of Stop Reagent (Sigma-Aldrich, Inc). The absorbance at 450 nm with reference wavelength at 630 was determined for each well by using Tecan Sunrise microplate reader with Magellan™ software (Tecan Austria GmbH).
